# Cognitive dysfunction in severe chronic obstructive pulmonary disease (COPD) with or without Long-Term Oxygen Therapy (LTOT)

**DOI:** 10.1186/s40248-015-0013-4

**Published:** 2015-04-19

**Authors:** Roberto W Dal Negro, Luca Bonadiman, Fernanda P Bricolo, Silvia Tognella, Paola Turco

**Affiliations:** National Centre for Respiratory Pharmacoeconomics and Pharmacoepidemiology – CESFAR, Verona, Italy; Lung Unit, Orlandi General Hospital, ULSS22 Regione, Veneto, Italy; Research & Clinical Governance, Verona, Italy

**Keywords:** Cognition impairment, COPD, Long term oxygen treatment, Severe chronic obstructive pulmonary disease

## Abstract

**Background:**

Chronic Obstructive Pulmonary Disease (COPD) is a progressive respiratory condition which can lead to comorbidities of variable severity, cognitive dysfunction included. The role of supplemental oxygen in preventing COPD-induced cognitive deterioration is still debated, but only episodically investigated. The aim of this study was to compare the cognitive pattern of hypoxemic COPD subjects treated with long-term oxygen (LTOT) to that of patients of comparable severity assuming oxygen on irregular basis, and to normal reference values.

**Methods:**

Lung function, arterial blood gases, health status, and cognitive function measured by means of four psychometric tests focusing different domains of cognition (such as: MMSE, Clock test; TMT-A; TMT-B) were assessed in 146 well matched hypoxemic COPD patients (males n = 96, 66%; mean age = 70.5 ± 12.9). Seventy-three patients were assuming long-term oxygen (LTOT), while the remaining seventy-three were only using oxygen as needed (AN). *Regarding statistics, t* test and ANOVA (Duncan test) were used to analyze data, assuming a p < 0.05 as the lowest limit of significance.

**Results:**

Even though all COPD patients showed a poorer psychometric profile vs corresponding normal reference values, LTOT patients showed a lower prevalence of severe deterioration in cognition. Also the extent of impairment was significantly lower in these patients when assessed by TMT-A and TMT-B (p < 0.012 and 0.001, respectively), but not when measured by MMSE and Clock test (both p = ns). Several domains of cognition are variably affected by persistent hypoxemia in COPD patients. A panel of psychometric tools is needed for identifying the pattern of cognitive dysfunctions in these patients. Memory and attention (functions assessed by MMSE and Clock test) are only mildly-moderately affected, while visual processing, reproduction of numeric sequences, cognition flexibility, and shifting capacity (functions assessed by TMT-A and TMT-B) are much more deteriorated (p < 0.012 and p < 0.001, respectively).

**Conclusions:**

Only LTOT allows to preserve significantly (p < 0.022) cognitive functions from the COPD-induced deterioration. This assumption is of strategic value for COPD patients who are prescribed long-term oxygen because they frequently are not aware of the cognitive risks related to their condition.

## Background

Chronic Obstructive Pulmonary Disease (COPD) is a pathological condition of respiratory system prevailing since the 5^th^ decade of life and characterized by a high socio-economic impact [1-3]. COPD can progressively affect the function of other organs (e.g. heart, vasculature, muscles, kidney, liver, gastro-enteric apparatus, and brain) leading to comorbidities of different severity [4,5].

Impairment of cognitive functions is one of the effects of comorbidities related to COPD [6-9]. It has long been associated with severe pulmonary dysfunction [10-15], even if at a changing prevalence in different studies, because depending on the methods for assessing cognition and on the consistency of subjects’ sample [16-20]. At present, some authors affirm that the lung function impairment assessed by means of FEV_1_ can be associated to a progressive cognitive function impairment in later life [15]. It has been demonstrated that hypoxemia *per se* can play a role in affecting cognition [21], and it might contribute to the onset and the progressive worsening of cognitive functions in subjects suffering from COPD, particularly in most severe cases [22]. Neverheless, other authors support the hypothesis that cognitive function is only mildly impaired without hypoxemia, and that cognitive dysfunction is higher in hypoxemic patients [9].

On the other hand, the role of supplemental oxygen in preventing the impairment of COPD-induced cognitive dysfunction is still debated.

Aim of the present study was to compare the cognitive dysfunction assessed in hypoxemic COPD subjects managed according to a strict protocol of home long-term oxygen (LTOT) to that of patients of comparable severity but assuming oxygen on irregular basis as needed (AN), and to normal reference values.

## Methods

The participants enrolled in the present study were patients suffering from hypoxic or hypoxemic/hypercapnic COPD as defined in the GOLD guidelines [23]. Half patients were managed according to a regular program of home LTOT, while the remaining 50% of subjects used only oxygen as needed, without any predefined protocol, and in the vast majority of cases it was due to their adamant refusal of LTOT.

Each patient was assessed for: lung function (complete spirometry using CPFS/D – Medical Graphics Co.; Oak Grove Parkway, St. Paul, Minnesota, USA); arterial blood gases (using the ABL 735 Analyzer, Radiometer, Copenhagen, DK); health status (using the COPD Assessment test [CAT] questionnaire) [24,25]; and disability (using the Medical Research Council [MRC] dyspnoea scale) [26]. The prevalence of comorbidities was also assessed in all patients by means of Charlson morbidity index (C.M.I.) [27], together with their smoking history.

Cognition was evaluated in each patient by means of four validated psychometric questionnaires focusing different cognitive domains and characterized by different sensitivity. They were sequentially administered to all patients [22]: (1) the Mini Mental Status test (MMSE), which assesses spatial and time orientation, attention and calculation skills (normal score: ≥ 27 points; moderate cognitive impairment: 24–18 points; severe cognitive impairment: < 18 points [28], (2) the Clock Drawning test, which assesses memory, attention and symbolic representation (normal score: 7–10 points; limit for cognitive impairment: ≤ 6 points) [29,30]; (3) the Trial Making test (TMT) A, which assesses visual processing and reproduction of numeric sequences (limit for cognitive impairment: ≥ 94 sec) [29,30]; (4) the TMT B, which assesses cognition flexibility and shifting capacity (limit for cognitive impairment: ≥ 283 sec) [30].

Psychometric data obtained from normal age-matched controls were used as reference values [21].

Written informed consent was obtained from all subjects participating in the study (n. study approval 3898/2011).

### Statistics

All data were expressed as means ± SD (standard deviation). Comparisons between groups were carried out by means of *t* test. Data of both groups were also compared to normal reference values by means of ANOVA (Duncan test). A p < 0.05 was accepted as the lowest limit of statistical significance.

## Results

The total sample consists of 146 subjects (males n = 96; 66%; mean age = 70.5 ± 12.9 years ) suffering from hypoxemic (and/or hypercapnic) COPD. Seventy-three patients (males n = 49, 67.1%; mean age = 71.2 ± 9.1) were managed according to a strict protocol of regular home LTOT with liquid oxygen for 2.9 years ± 0,9, with a mean oxygen use of 1.7 L/min ± 0.6, for > 15 hours/day, whereas the remaining 73 patients (males n = 48; 65.7%%; mean age = 70.9 ± 8.9 years) used oxygen only as needed, on irregular basis.

The general characteristics of both groups are reported in Table 1. The two groups proved well matched regarding age, gender, smoking history, BMI, dyspnoea score, arterial blood gases, and lung function (all *t* test comparisons p < 0.05). Also the mean C.M.I. was absolutely comparable in the two groups, being cardiovascular and metabolic comorbidities the most represented (such as 67.4%) and equally prevailing in both groups.

**Table 1:** **General characteristics of patients at baseline (means ± SD) and their statistical comparison (** ***t*** **test)**

	**without LTOT (n = 73)**	**with LTOT (n = 73)**	**p**
**age** (ys)	70.9 ± 8.9	71.2 ± 9.1	ns
**FEV**_**1**_ % pred.	40.2 ± 10.3	41.6 ± 11.1	ns
**FEV**_**1**_ **/FVC**	437 ± 9.2	44.2 ± 10.2	ns
**BMI**	27.5 ± 5.1	27.9 ± 6.4	ns
**Active smokers**	9.6%*	6.8%*	ns
**Former smokers**	69.9%*	72.6%*	ns
**PaO**_**2**_ (mmHg)	53.3 ± 7.9	54.2 ± 8.3	ns
**PaCO**_**2**_ (mmHg)	45.8 ± 9.9	46.1 ± 10.2	ns
**CAT** score	19.9 ± 4.9	18.8 ± 5,6	ns
**MRC** scale	2.8 ± 0.7	2.7 ± 0.8	ns
**C.M.I.**	4.2 ± 2.0	4.3 ± 1.9	ns

*Smoking history is indicated in % active or former smokers.

**Figure 1:**
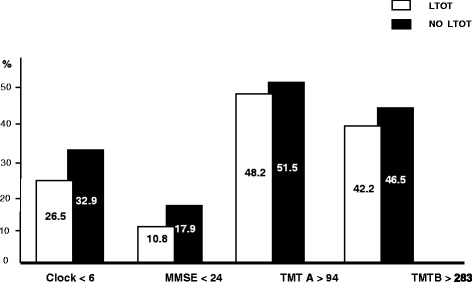
The prevalence of severe cognition impairment in the two groups of patients by different investigational tools.

The prevalence of severe cognitive impairment assessed in the two groups is reported in Figure 1. LTOT patients showed a significant lower prevalence of severe cognitive deterioration, independently of the psychometric questionnaire used for measurements. In other words, all domains of cognition were significantly more deteriorated in AN patients.

Also the absolute extent of cognitive dysfunction was measured in both groups of patients, and corresponding data are reported in Table 2, together with the significance level of their statistical comparison. In particular, while the Clock test and the MMSE scores were equally informative in the two groups, the TMT-A and the TMT-B scores proved significantly different, and LTOT patients resulted significantly less impaired in their cognition compared to AN patients (p < 0.012 and p < 0.001, respectively) (Table 2).

**Table 2:** **Extent (mean ± SD) of cognition impairment assessed in the two groups and significance of their comparison (** ***t*** **test)**

**test**	**LTOT (n = 73)**	**NO LTOT (n = 73)**	**p**
Clock test < 6	4.2 ± 1.7	3.9 ± 1.9	ns
MMSE < 24	20.1 ± 3.1	22.0 ± 2.3	ns
TMT-A > 94	132.2 ± 35.8	155.3 ± 52.5	0.012
TMT-B > 283	322.1 ± 36.2	344.2 ± 31.8	0.001

**Figure 2:**
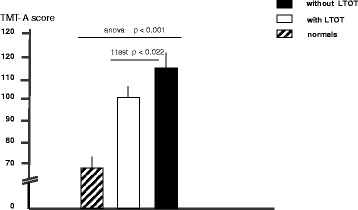
Extent of cognition impairment measured by the TMT-A test in patients with and without LTOT, and in normal subjects (all aged 65–75 ys) (*t* test between groups and ANOVA among groups).

**Figure 3:**
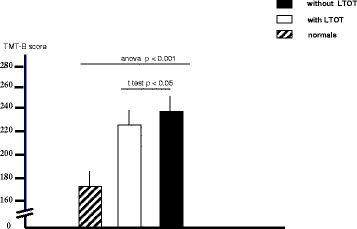
Extent of cognition impairment measured by the TMT-B test in patients with and without LTOT, and in normal subjects (all aged 65–75 ys) (*t* test between groups and ANOVA among groups).

TMT-A and TMT-B proved the most discriminant psychometric tools and those worth of further analysis. Currently, as normal reference values for subjects aged 40–79 years are only available in the literature for the TMT-A and TMT-B [18], TMT-A and TMT-B scores obtained in the present study (range of age 65–75 years) were compared to corresponding normal reference values (Figures 2 and 3).

When compared to normals, the extent of cognitive dysfunction was significantly higher in all patients when assessed by TMT- A and TMT-B (ANOVA p < 0.001 for both comparisons). Nevertheless, LTOT patients proved significantly less limited in their cognition than AN patients (*t* test p < 0.022 and p < 0.05, respectively).

## Discussion

It is increasingly accepted that COPD is an inflammatory multicomponent, pathological condition which can affect several functions of different organs. Cognition represents one of these aspects, even if little attention has been paid to the effects of COPD on cognition in clinical practice, particularly in the presence of hypoxemia.

In general, the occurrence of significant limitations in cognitive functions was found to be associated with severe pulmonary dysfunction, particularly when chronically persisting, as in the case of COPD [12-15]. Recently, a specific study focused on these aspects and confirmed that both the prevalence and the extent of cognitive deterioration occurring in subjects with stable COPD of different severity are directly related to the severity of their pulmonary condition, hypoxemia included. In these cases, several domains of cognition are involved, and then several functions can be deteriorated, even substantially [22].

Hypoxemia has long been described as able to affect memory [21], even if this condition has been recently regarded as unlikely to account *per se* for the cognitive dysfunction in COPD [9]. On the other hand, its crucial role has been emphasized by other investigations which recently proved that the chronic persistence of hypoxemia is directly related to the extent of cognition impairment when investigated by means of the Montreat Cognitive Assessment [31]. Moreover, perception, attention, and short term memory has been described as significantly impaired in hypoxemic COPD patients in stable conditions, and the cause of this cognitive impairment has been related to neurophysiological events, such as the sustained presence of a decreased prefrontal cortex circulation [32].

Furthermore, also the intermittent, even if persistently occurring, hypoxemia which characterizes the obstructive sleep apnea syndrome (OSAS) has been suggested to have a role in developing cognitive impairment at variable degrees, particularly in ageing population (i.e. attention, episodic memory, working memory) [33].

When compared to corresponding normal reference values, a substantial deterioration in cognition was assessed in all COPD patients in present study, but according to a peculiar pattern. In particular, some cognitive functions resulted only mildly-moderately impaired (such as, those assessed by MMSE and Clock test), while others (such as, those assessed by TMT-A and TMT-B) resulted much more deteriorated. It would then be surmised that several domains of cognition are variably affected by persistent hypoxemia in COPD patients, such as: memory, attention, symbolic representation and visual processing, reproduction of numeric sequences, cognition flexibility, and shifting capacity. Long-term oxygen is presumed to consent a slower deterioration of all these cognitive functions. Some of them (i.e. memory and attention) might be improved independently of the duration of oxygen assumption, while others (such as: visual processing, reproduction of numeric sequences, cognition flexibility, and shifting capacity), that likely are the most complex functions in neuro-psychological terms, are only sensitive to long-term oxygen treatment.

Consequently, a large panel of investigational instruments, covering a wide range of cognitive domains and differently sensitive is needed in order to specify the psycometric profile of these patients. Likely this is the reason why the occurrence of cognitive dysfunction was usually regarded as variable and depending on the psychometric test used [34,35].

This particular aspect is also crucial when comparing the effect of different strategies of oxygen treatment (such as: regular OTLT vs oxygen as needed) on cognitive dysfunction. In the present study, the commonly used MMSE and Clock Drawing Test failed to discriminate the effects of home LTOT on cognition from those of oxygen as needed. Only TMT-A and TMT-B showed that long-term oxygen was much more effective in preserving these patients from cognitive dysfunctions, even if they still remain substantially limited compared to normal individuals. Consequently, different instruments provided with specific sensitivities should be used in order to highlight and differentiate the pattern of response to oxygen treatment.

The hypothesis that hypoxemia-induced cognitive deterioration might depend on complex neuro-physiological mechanisms in COPD (i.e. systemic inflammation and vascular damage affecting brain performance) seems to be confirmed by a study carried out according to a multiparametrical approach (such as by means of Short Test of Mental Status, transcranial Doppler ultrasonography, five cardiovascular tests, and a questionnaire of autonomic function) in a small sample of hypoxemic COPD patients [36]. Also the effect of a three-month LTOT on the function of central and autonomic nervous system was investigated in this study. Patients’ cognition was shown to improve proportionally to the amelioration of their neurological parameters, cerebral blood flow velocity, and autonomic function following LTOT, thus emphasizing the reversibility (at least partial) of the COPD-induced cognitive deterioration and the therapeutic effect of long-term oxygen from this point of view.

**Figure 4:**
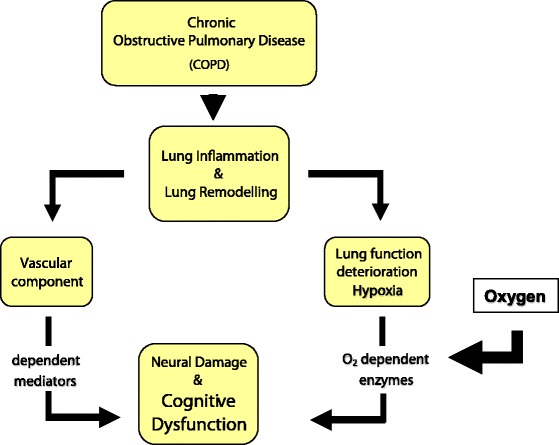
Deterioration of cognition in Chronic Obstructive Airway Disease: a comprehensive vision of the oxygen role in modulating the pathways of cognitive dysfunction.

Persistent inflammation underlies chronic airflow limitation and is maximally effective in the severest conditions. As cognitive deterioration has been proved to be related to hypoxemia and to severity of airway obstruction [22], the active role of chronic inflammation in sustaining cognitive dysfunction can also be suggested in COPD. A comprehensive vision of the role of hypoxemia and of oxygen supplementation in modulating the pathways of cognitive dysfunction is reported in Figure 4.

In clinical terms, the strong evidence that cognition may be significantly deteriorated in hypoxemic COPD subjects contributes to explain why these subjects frequently demonstrate an insufficient self management and their adherence to therapeutic strategies frequently results inadequate. The psychometric profile of these subjects, as shown in the present study, can also contribute to explain their frequent requests for admission to health institutions and the consequent high economic impact.

## Conclusions

In conclusion, focusing the effect of COPD on cognition is a relevant issue for all COPD patients, independently of their severity. The assumption that hypoxemic COPD subjects have a substantial cognitive dysfunction mainly affecting peculiar aspects of cognition is of strategic value for those COPD patients who are prescribed long-term oxygen because they frequently are not aware of the systemic risks related to their condition. They should be increasingly encouraged to accept LTOT, when needed, because this therapeutic strategy is effective in preserving their cognitive function from the deterioration induced by their chronic respiratory limitations, and in preventing progression of cognitive dysfunction.
